# DIAGNOSTIC ACCURACY OF BARIUM ENEMA FINDINGS IN HIRSCHSPRUNG'S DISEASE

**DOI:** 10.1590/0102-6720201600030007

**Published:** 2016

**Authors:** Mehran PEYVASTEH, Shahnam ASKARPOUR, Nasrollah OSTADIAN, Mohammad-Reza MOGHIMI, Hazhir JAVAHERIZADEH

**Affiliations:** 1 Department of Pediatric Surgery, Imam Khomeini Hospital, Iran; 2 Nursing Care Research Center in Chronic Diseases and Department of Pediatric Gastroenterology, Ahvaz Jundishapur University of Medical Sciences, Ahvaz, Iran

**Keywords:** Hirschsprung's disease, Chronic constipation, Barium enema

## Abstract

**Background:**

Hirschsprung's disease is the most common cause of pediatric intestinal obstruction. Contrast enema is used for evaluation of the patients with its diagnosis.

**Aim:**

To evaluate sensitivity, specificity, positive predictive value, and negative predictive value of radiologic findings for diagnosis of Hirschsprung in patients underwent barium enema.

**Methods:**

This cross sectional study was carried out in Imam Khomeini Hospital for one year starting from 2012, April. Sixty patients were enrolled. Inclusion criteria were: neonates with failure to pass meconium, abdominal distention, and refractory constipation who failed to respond with medical treatment. Transitional zone, delay in barium evacuation after 24 h, rectosigmoid index (maximum with of the rectum divided by maximum with of the sigmoid; abnormal if <1), and irregularity of mucosa (jejunization) were evaluated in barium enema. Biopsy was obtained at three locations apart above dentate line. PPV, NPV, specificity , and sensitivity was calculated for each finding.

**Results:**

Mean age of the cases with Hirschsprung's disease and without was 17.90±18.29 months and 17.8±18.34 months respectively (p=0.983). It was confirmed in 30 (M=20, F=10) of cases. Failure to pass meconium was found in 21(70%) cases. Sensitivity, specificity, PPV, and NPV were 90%, 80%, 81.8% and 88.8% respectively for transitional zone in barium enema. Sensitivity, specificity, PPV, and NPV were 76.7%, 83.3%, 78.1% and 82.1% respectively for rectosigmoid index .Sensitivity, specificity, PPV, and NPV were 46.7%, 100%, 100% and 65.2% respectively for irregular contraction detected in barium enema. Sensitivity, specificity, PPV, and NPV were 23.3%, 100%, 100% and 56.6% respectively for mucosal irregularity in barium enema.

**Conclusion:**

The most sensitive finding was transitional zone. The most specific findings were irregular contraction, mucosal irregularity, and followed by cobblestone appearance.

## RESUMO

**Racional:**

A doença de Hirschsprung é a causa mais comum de obstrução intestinal pediátrica. Enema baritado é usado para a avaliação dos pacientes com o diagnóstico .

**Objetivo:**

Avaliar a sensibilidade, especificidade, valor preditivo positivo e valor preditivo negativo de achados radiológicos para diagnóstico de Hirschsprung em pacientes submetidos ao enema opaco.

**Métodos:**

Este estudo transversal foi realizado em Imam Khomeini Hospital por um ano a partir de abril de 2012. Sessenta pacientes foram incluídos. Os critérios de inclusão foram: recém-nascidos com insuficiência de passagem de mecônio, distensão abdominal, e constipação refratária sem resposta ao tratamento médico. Foram avaliadas no enema zona de transição, atraso na evacuação de bário após 24 h, índice retossigmoide (máximo do diâmetro do reto dividido pelo máximo do sigmóide; anormal se <1), e as irregularidades da mucosa (jejunização). Biópsia foi obtida em três localizações acima da linha dentada. VPP, VPN, especificidade e sensibilidade foram calculados para cada achado.

**Resultados:**

A idade média dos casos com a doença de Hirschsprung e sem foi 17,90±18,29 meses e 17,8±18,34 meses, respectivamente (p=0,983). Confirmou-se em 30 (M=20, F=10) dos casos. Falha no mecônio foi encontrada em 21 (70%) casos. Sensibilidade, especificidade, VPP e VPN foram de 90%, 80%, 81,8% e 88,8%, respectivamente, para a zona de transição no enema. Sensibilidade, especificidade, VPP e VPN foram 76,7%, 83,3%, 78,1% e 82,1%, respectivamente para o índice de retossigmoide. Sensitividade, especificidade, VPP e VPN foram 46,7%, 100%, 100% e 65,2%, respectivamente, para contração irregular detectada no enema baritado. Sensibilidade, especificidade, VPP e VPN foram de 23,3%, 100%, 100% e 56,6%, respectivamente, para a irregularidade da mucosa.

**Conclusão:**

O achado mais sensível foi zona de transição. Os achados mais específicos foram contração irregular, irregularidade da mucosa, e seguido por aparecimento de mucosa em forma de paralelepípedos.

## INTRODUCTION

Hirschsprung's disease (HD) is a common cause of pediatric intestinal obstruction^9^. It is caused by the failure of the ganglion cells to migrate cephalocaudally through the neural crest causing absence of ganglion cell in all or some parts of colon^1^. Prevalence of disease was reported about 1:5000 live birth and male to female ratio: 4/1^4^ ^,^ ^11^. Hirschsprung's disease was reported as the etiology of childhood bowel obstruction in about 12% of cases in our country^15^. In another study from Nigeria, it is the etiology of intestinal obstruction in children with a frequency about 13.85%^14^.

Although the initial diagnosis is mainly based on clinical history and examination and followed by pathological examination^3^, radiographic contrast evaluation may be useful in diagnosis^19^. Anorectal manometry, rectal suction biopsy, and barium enema are used in our country. Anorectal manometry is not available in many hospitals. Barium enema (BE) is available in many centers even without pediatric surgeon. 

So, the aim of this study was to evaluate sensitivity, specificity, positive predictive value, and negative predictive value of radiologic findings for diagnosis of HD in patients underwent barium enema.

## METHODS

This study was approved by Ethical Committee of the Ahvaz Jundishapur University of Medical, Ahvaz, Iran.

This cross sectional study was carried out in Imam Khomeini Hospital. Sixty patients were enrolled. Duration of study was one year starting from 2012 April. Inclusion criteria were: neonates with delayed meconium passage and clinical symptoms of Hirschsprung (i.e., failure to pass meconium, constipation, and abdominal distention); and children with refractory constipation who failed to respond with medical treatment.

Children with history of anorectal surgery, without follow up were excluded. Informed consent was signed by all parents before inclusion. All patients underwent barium enema and full thickness rectal biopsy. Barium enema was done under supervision of experienced radiologists who are familiar with pediatric radiology. 

**FIGURE 1:**
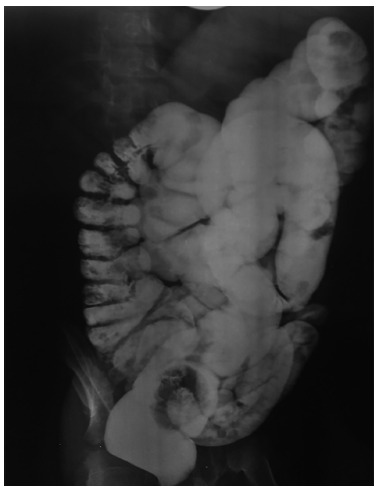
Delayed passage of contrast material was seen after 48 h of barium enema: RI was normal; IC was not seen

**FIGURE 2:**
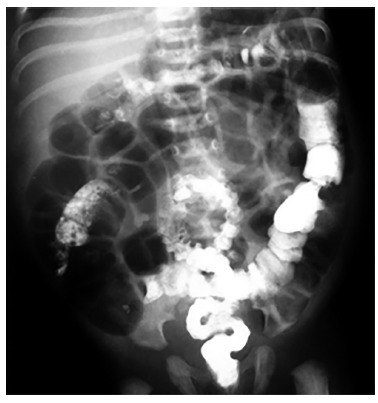
Abdominal distention and irregular contraction

Following findings were evaluated in BE of each patient: transitional zone (TZ), delay in barium evacuation after 24 h, rectosigmoid index (RI), mucosal irregularity (jejunization), cobblestone appearance, and irregular contraction (Figures 1 and 2). 

Rectosigmoid index is obtained by dividing the widest diameter of the rectum by the widest diameter of the sigmoid loop when the colon is fully distended by the contrast medium^5^ ^,^ ^16^. The normal rectosigmoid index is ≥1. In standard length HD the recosigmoid index is ≤ 1.

Full thickness biopsy, as a classic approach^4^, was obtained by an experienced pediatric surgeon at three location (2, 5, and 7 cm) apart above dentate line. Proximal and distal ends of biopsy specimens were marked for pathologist. PPV, NPV, specificity and sensitivity was calculated for each finding according to the full thickness biopsy as gold standard.

### Statistical analysis

Chi-square was used for analysis. P value <0.05 was considered significant. SPSS (Chicago, IL, USA) version 13.0 was used for analysis. 

## RESULTS

Mean age of the cases with HD and without was 17.90±18.29 (range 1-60 months) and 17.8±18.34 (range 1-60 months) months respectively (p=0.983). Minimum and maximum of age was one and 60 months in both groups. From all cases, abdominal distention was present in 23 (76.7%); failure to pass meconium was noted in 21 (70%); and constipation in 12 (40%). HD was confirmed using full thickness rectal biopsy in 30 (M=20, F=10) of cases. Of 30 normal subjects, 23 were male and seven women (p=0.390). Of 30 patients with HD, 17 were =/< 1 years old. Frequency of TZ findings in BE is shown in Table 1. Sensitivity, specificity, PPV, and NPV were 90% (95% CI: 73.44-97.77%); 80.00% (95% CI: 61.42%-92.24%), 81.82% (95% CI: 64.53-92.98%); and 88.89% (95% CI: 70.81-97.52%) respectively for TZ in barium enema. 

**TABLE 1:** Frequency of TZ findings on BE in subjects with and without HD

	HD(+)	HD(-)
TZ(+)	27(90%)	6(20%)
TZ(-)	3(10%)	24(80%)

TZ=transitional zone; HD=Hirschsprung's disease

Frequency of RI finding in BE among subjects with and without HD is shown in Table 2. Sensitivity, specificity, PPV, and NPV were 76.67% (95% CI: 57.71%-90.02%) ,83.33% (95% CI: 65.27%-94.30%); 82.14% (95% CI: 63.09%-93.87%), and 78.12% (95% CI: 60.02%-90.68%) respectively for RI. 

**TABLE 2:** Frequency of RI finding on BE among subjects with and without HD

	HD(+)	HD(-)
RI(+)	23(76.7%)	5(16.7%)
RI(-)	7(23.3%)	25(83.3%)

RI=rectosigmoid index

Frequency of irregular contractions (IC) finding among cases with and without HD is shown in Table 3. Sensitivity, specificity, PPV, and NPV were 46.67% (95% CI: 28.36%-65.66%); 100% (95% CI: 88.32%-100%);100% (95% CI: 76.66%-100%); and 65.22% (95% CI=49.75%-78.94%) respectively for irregular contraction detected in barium enema.

**TABLE 3:** Frequency of IC in BE among subjects with and without HD

	HD(+)	HD(-)
IC(+)	14(46.7%)	0(0)
IC(-)	16(53.3%)	30(100%)

HD=Hirschsprung's disease; BE=barium enema; IC=irregular contraction

Frequency of mucosal irregularity in BE in subjects with and without HD is shown in Table 4. Sensitivity, specificity, PPV, and NPV were 23.33% (95% CI: 9.98%-42.29%); 100% (95% CI: 88.32%-100%);100% (95% CI: 58.93%-100%); and 56.6% (95% CI: 42.28%-70.16%) respectively for mucosal irregularity in barium enema.

**TABLE 4:** Frequency of mucosal irregularity in barium enema among subjects with and without HD

	HD(+)	HD(-)
Mucosal irregularity(+)	7(23.3%)	0(0)
Mucosal irregularity(-)	23(76.7%)	30(100%)

HD=Hirschsprung's disease

Frequency of cobblestone appearance is shown in Table 5. Sensitivity, specificity, PPV, and NPV were 13.3% (95% CI: 3.84%-30.74%); 90% (95% CI: 73.44%-97.77%); 57.14% (95% CI: 18.75%-89.58%); and 50.9% (95% CI: 36.84%-64.43%) respectively for cobblestone appearance in barium enema.

**TABLE 5:** Frequency of cobblestone finding in BE among subjects with and without HD

	HD(+)	HD(-)
Cobblestone(+)	4(13.3%)	3(10%)
Cobblestone(-)	26(86.7%)	27(90.0%)

HD=Hirschsprung's disease

**TABLE 6:** Summary of sensitivity and specificity of radiologic findings in HD

Finding	Specificity	Sensitivity
TZ	80.00%(95%CI:61.42%-92.24%)	90%( 95% CI: 73.44-97.77%)
RI	83.33%(95%CI:65.27%-94.30%)	76.67% (95% CI: 57.71%-90.02%)
Cobblestone	90%(95%CI:73.44%-97.77%)	13.3%(95%CI:3.84%-30.74%)
Mucosal irregularity	100%(95%CI=88.32%-100%)	23.33%(95%CI:9.98%-42.29%)
IC	100%(95%CI:88.32%-100%)	46.67%(95%CI:28.36%-65.66%)

TZ=transitional zone; RI=rectosigmoid index; IC=irregular contraction

## DISCUSSION

In this study of 60 cases of HD was confirmed using biopsy in 30 cases. Of 30 cases with HD, 17 were ≤ 1 year. In another study, the majority of the patients presented after the first year of life^10^.

Of HD cases, 76.67% had inverted rectosigmoid index in barium enema. In Garcia et al. RI was positive in 79% of cases with HD^7^. Alehossein et al^2^ reported inverted rectosigmoid index among 86% of children with HD that was slightly higher than Garcia et al^7^ and our study.

Failure to pass meconium was noted in 21 (70%) of children with HD. In another study, 72.2% of children with HD had delayed meconium passage^2^. The result of the two studies were similar.

Mucosal irregularity (jejunization) was found in 7 (23.3%) cases. Mucosal irregularity was seen in 7 (21%) with HD in Alehossein et al study^2^. Irregularity of mucosa was not found in children without HD in this study and Alehossein et al.´s^2^. The result of two studies were similar.

Sensitivity, specificity, PPV, and NPV were 13.3%,90%, 57.1%, and 50.9% respectively for cobblestone appearance in barium enema. Sensitivity, specificity, PPV, and NPV of cobblestone appearance were 18.3%, 94.7%, 76.5%, and 36.5 in Alehossein et al study^2^. O'Donovan et al. referred sensitivity and specificity of cobblestone appearance in 5% and 100%, respectively^13^.

IC was found in 46.7% of children with HD. Which was similar to Alehossein et al study who found IC in 43%^2^. IC was not negative in children with HD in the current and Alehossein and colleagues papers^2^.

 In this study, 90% had TZ in barium enema. Pratap et al. related that in proven HD, 89% had TZ in barium enema^17^. In Alehossein et al. paper, TZ was positive in 94% of children with HD^2^. Garcia et al. related that TZ agreed with histopathologic index in 87% of cases^7^. In the study of Noviello and colleagues of 18 cases aged <1 year, three had TZ in barium enema and rectal suction biopsy confirmed HD in nine^12^. Taxman et al. ^20^ analyzing 58 constipated infants and children who underwent rectal suction biopsy, 8% of children with aganglionosis had TZ in barium enema which was comparable to our study. Here in cases without HD, 20% had TZ in barium enema. In Diamond et al paper, 45% of subjects without HD showed TZ in barium enema^6^.

We used barium study in our hospital. It was due to some economic limitation for our patients. Anorectal manometry was not available due to some limitation. In de Lorjin and colleagues^5^ research, sensitivity of rectal suction biopsy, anorectal manometry, and contrast enema were 93%, 83%, and 76% respectively. Specificity of rectal suction biopsy, anorectal manometry and contrast exam were 100%, 93%, and 97% respectively. They showed no significant difference among values^5^.

Previous studies^8^ ^,^ ^18^ showed that TZ and RI were the most frequent sign in contrast enema which was similar to our study.

Another multicenter research is recommended to evaluate diagnostic accuracy of barium enema in low resource setting.

## CONCLUSION

Mucosal irregularity and irregular contraction were the most specific radiologic findings with the specificity about 100%. Transitional zone was the most sensitive radiologic finding with the sensitivity about 80%.
